# Identifying Biomarkers Associated with Venous Infarction in Acute/Subacute Cerebral Venous Thrombosis

**DOI:** 10.14336/AD.2020.0405

**Published:** 2021-02-01

**Authors:** Jiangang Duan, Xinyi Leng, Ziping Han, Yanning Cai, Chunxiu Wang, Gary Rajah, Haiqing Song, Yuchuan Ding, Xunming Ji

**Affiliations:** ^1^Department of Emergency, Xuanwu Hospital, Capital Medical University, Beijing, China.; ^2^Department of Medicine & Therapeutics, Chinese University of Hong Kong, Hong Kong SAR, China.; ^3^Cerebrovascular Diseases Research Institute, Xuanwu Hospital, Capital Medical University, Beijing, China.; ^4^Department of Neurobiology, Xuanwu Hospital, Capital Medical University, Beijing, China.; ^5^Department of Evidence-based Medicine, Xuanwu Hospital, Capital Medical University, Beijing, China.; ^6^Department of Neurosurgery, Wayne State University School of Medicine, Detroit, Michigan, USA.; ^7^Department of Neurology, Xuanwu Hospital, Capital Medical University, Beijing, China.

**Keywords:** biomarkers, cerebral venous infarction, cerebral venous thrombosis

## Abstract

Among cerebral venous thrombosis (CVT) patients, those with venous infarction have more severe clinical presentations and worse outcomes. Identifying biomarkers associated with venous infarction in CVT may help understand the pathogenesis and provide potentially useful therapeutic markers. Fifty-two CVT patients were prospectively recruited and divided into three groups: acute/subacute CVT with venous infarction (ASVI, n=30), without venous infarction (ASOVI, n=13), and chronic CVT (n=9). Blood brain barrier (BBB) permeability-related proteins, including claudin-5, occludin, matrix metalloproteinase-9, glial fibrillary acidic protein, and S100B, and inflammation-related factor high-sensitivity C-reactive protein (hs-CRP), were tested in serum and/or cerebrospinal fluid upon admission. We compared these biomarkers between the three groups and investigated their associations with venous infarction and clinical symptom severity in acute/subacute CVT patients on admission using the NIH Stroke Scale (NIHSS). Serum hs-CRP was significantly higher in acute/subacute CVT patients than chronic CVT patients. For acute/subacute CVT patients, levels were significantly higher in the ASVI group than the ASOVI group for serum claudin-5 (medians 2.80 vs. 2.50 mg/I, respectively, P = 0.039) and hs-CRP (medians 17.25 vs. 2.27 mg/l, respectively, P = 0.003). Both these biomarkers, analyzed as categorical or continuous variables, were also significantly associated with venous infarction in acute/subacute CVT patients after logistic regression analysis. Additionally, hs-CRP was positively correlated with the NIHSS (*r* = 0.710, P < 0.001) on admission in acute/subacute CVT patients. In CVT patients, venous infarction was associated with BBB disruption and potentially inflammation. Hs-CRP might serve as a biomarker reflecting the clinical severity of CVT in the acute/subacute stages.

Studies using conventional or novel imaging methods have suggested that cerebral venous infarction is not uncommon; it occurs in approximately 60% of patients with cerebral venous thrombosis (CVT) [1-3]. In acute/subacute CVT patients, the presence of venous infarction is associated with more severe clinical presentations and worse outcomes [4-5]. The underlying pathophysiology of venous infarction in the presence of CVT remains controversial despite clinical and experimental studies [6]. Identifying biomarkers associated with venous infarction and clinical severity in CVT patients may help in understanding the pathophysiology of venous infarction and may provide novel therapeutic markers for preventing and/or treating venous infarction in the future.

Disruption of the blood-brain barrier (BBB) is generally considered to be associated with venous infarction [7]. BBB permeability is modulated by protein-protein interactions in tight junctions (TJs) [8], of which claudin-5 and occludin are the main components [9,10]. In addition, plasma matrix metalloproteinase-9 (MMP-9) is reported to be associated with the degradation of certain TJs in laboratory studies and has been correlated with the severity of hemorrhagic transformation after cardioembolic stroke [11,12]. S100 calcium binding protein B (S100B) may also play a critical role in maintaining the integrity of the BBB and cellular interactions in the neurovascular unit [13,14] and has been used as a marker for brain damage and BBB disruption [15]. Moreover, enhanced glial fibrillary acidic protein (GFAP) levels have been associated with BBB impairment [16].

However, our latest research suggested that inflammation might develop after CVT and is significantly correlated with the severity of clinical symptoms on admission and poor outcomes at discharge [17]. A recent study also reported that C-reactive protein (CRP), a marker of acute inflammation, had predictive value for venous thromboembolism [18]. High-sensitivity CRP (hs-CRP), a reliable inflammatory biomarker, can accurately detect low-grade inflammation and is routinely tested in clinical practice [19]. Furthermore, an experimental study has revealed that CVT-induced BBB disruption and venous infarction may depend on inflammatory cell recruitment [20].

Therefore, the proteins related to BBB permeability and inflammation may be elevated in the serum and/or cerebrospinal fluid (CSF) in CVT patients with venous infarction. Moreover, inflammation may also play an important role in determining the severity of clinical symptoms.

Therefore, to better understand the pathophysiology and associated biomarkers of venous infarction in CVT, we tested the levels of biomarkers related to inflammation and BBB integrity in the serum and/or CSF of consecutively recruited CVT patients and investigated their association with venous infarction and clinical symptom severity on admission.

## MATERIALS AND METHODS

### Study population

The study protocol was approved by the Institutional Review Board of Xuanwu Hospital, Capital Medical University, Beijing, China. Consecutive patients with probable CVT admitted to Xuanwu Hospital between July 2015 and December 2016 were prospectively screened and recruited for this study. All participants or their legally authorized representatives provided informed consent. Data supporting the findings of this study are available from the first author upon reasonable request.

The inclusion criteria were as follows: (1) aged > 18 years; (2) consented to participate in the study and undergo lumbar puncture, conventional magnetic resonance imaging (MRI), MR black-blood thrombus imaging (MRBTI), and relevant blood tests on admission; and (3) diagnosed with acute, subacute, or chronic CVT by clinical, laboratory, and imaging examinations [21-23]. The exclusion criteria were as follows: (1) unknown interval between symptom onset and admission; (2) contraindications to MRI; (3) inability to provide informed consent; (4) significant liver or kidney dysfunction; or (5) traumatic brain injury or acute ischemic stroke on admission.

### Definition of CVT, venous infarction, and patient groups

CVT was diagnosed by experienced neurologists based on clinical presentations (e.g., increased intracranial pressure attributable to impaired venous drainage or focal brain injury from venous ischemia/infarction or hemorrhage) and cerebral imaging confirmation (conventional MRI and MRBTI sequence) [21-23]. Uncertainties in the diagnosis were resolved by agreement between two neurologists or consultation with a senior neurologist.

CVT is generally considered acute (0-7 days), subacute (8-15 days), or chronic (>15 days) based on the interval between symptom onset and admission. Additionally, the presence of thrombus signals in conventional T1- or T2-weighted sequences, contrast-enhanced MRI, and novel MRBTI [22-24] are used. However, the initial symptoms of CVT are sometimes subtle and non-specific, and acute and subacute thrombus signals often co-exist on MR images in the same patient, so the time of CVT onset cannot always be accurately determined. Therefore, we did not differentiate between acute and subacute CVT patients in our analyses. Venous infarction was defined as the presence of brain parenchymal lesions, including local edema and petechial or confluent cerebral hemorrhage, caused by occlusion of the cerebral veins or sinus on MRI [25]. All CVT patients were then classified into 3 groups according to the stage of CVT and presence of venous infarction: the acute/subacute CVT with venous infarction (ASVI) group, the acute/subacute CVT without venous infarction (ASOVI) group, and the chronic CVT group, which included those with and without venous infarction.

### Baseline data collection

We collected demographic data, the interval from symptom onset to admission, and disorders of consciousness including somnolence, stupor, coma, and delirium. We also assessed the severity of clinical symptoms on admission using the NIH Stroke Scale (NIHSS).

Genetic prothrombotic states, including antithrombin III deficiency, protein C and protein S deficiency, and homocysteinemia caused by mutations in the methylenetetrahydrofolate reductase gene were recorded. Acquired prothrombotic states, including nephrotic syndrome, oral contraceptive usage, antiphospholipid antibody syndrome, hyperhomocysteinemia, pregnancy, puerperium, hyperthyroidism, and cancer were also recorded. Data were collected on hematologic diseases including primary thrombocythemia and anemia, current craniocerebral infection (e.g., mastoiditis and intracranial infection), and current non-infectious inflammatory diseases such as systemic lupus erythematosus.

### Cerebrospinal fluid and blood tests

All patients received a routine lumbar puncture and peripheral blood collection upon hospital admission, before the initiation of standard treatments such as heparinization. Serum and CSF samples were processed by centrifugation at 100 *g* for 20 min and stored at -80 °C before analysis. All samples except hs-CRP were assayed by ELISA within one year of storage.

Hs-CRP levels were measured using a particle-enhanced immunoturbidimetric assay. Other reagents and samples were processed for testing at room temperature without additional heating. Before pipetting, samples were mixed thoroughly by gentle swirling to avoid foaming. Levels of claudin-5, occludin, and MMP-9 were measured from both the serum and CSF, while levels of GFAP, S100B, and hs-CRP were measured only from the serum, since the CSF sample volumes were insufficient for testing. Claudin-5, occludin, and MMP-9 levels were assayed using the following commercial ELISA kits respectively: LS-F8302 (LifeSpan, Seattle, WA, USA), LS-F12208 (LifeSpan), and Quantikine DMP900 (R&D Systems, WA, USA). GFAP and S100B levels were assayed using the GFAP ELISA kit (RD192072200R, Biovendor, Brno, Czech Republic) and the S100B ELISA kit (RD192090100R, BioVendor), respectively.

### Statistical analysis

Continuous variables are presented as medians (interquartile range, IQR). Categorical variables are presented as numbers (percentages). The baseline characteristics and levels of hs-CRP, claudin-5, occludin, and MMP-9 in the serum or CSF were compared between the three groups and between the ASVI and ASOVI groups, using the *χ*^2^ test for categorical variables and the Mann-Whitney test or the Kruskal-Wallis test for continuous variables. For biomarkers with P < 0.05 in univariate comparisons between ASVI and ASOVI patients, areas under receiver operating characteristic (ROC) curves (AUCs) were used to assess the capability, and the cut-off values, of the biomarkers to discriminate ASVI from ASOVI. The AUCs of different ROC curves were compared using Z tests.

Univariate logistic regression analyses were performed to determine the associations between the presence of venous infarction in acute/subacute CVT patients and variables including age, sex, current craniocerebral infection, acquired prothrombotic and genetic thrombotic states, and biomarkers with a P value < 0.05 in univariate comparisons between ASVI and ASOVI patients. Results are expressed as the odds ratios (ORs) with 95% confidence intervals (CIs). Relevant biomarkers were analyzed as categorical variables (dichotomized by optimal cut-off values from ROC analyses as shown above) and as continuous variables for logistic regression analyses.

Additionally, for the acute/subacute CVT patients, the Spearman correlation coefficient was used to assess linear relationships between the biomarkers and the severity of clinical symptoms on admission using the NIHSS.

All data analyses were performed using SPSS version 20.0 (SPSS, IBM, Armonk, NY, USA). A two-tailed P-value < 0.05 was considered statistically significant.

## RESULTS

### Study population

Overall, 87 consecutive patients with probable CVTs on admission were assessed for eligibility, of which 35 were excluded: 10 did not undergo an MRI examination due to contraindications or critical conditions, 15 were younger than 18 years old, 5 did not consent to lumbar puncture, 3 had significant liver or kidney dysfunction, and 2 had traumatic brain injuries. In the end, 52 patients with CVT were recruited, 30 in the ASVI group, 13 in the ASOVI group, and 9 in the CVT group (3 with venous infarction and 6 without). The median intervals from CVT onset to admission were 10, 15, and 40 days, in the ASVI, ASOVI, and chronic CVT groups, respectively. There was no significant difference in baseline characteristics between the ASVI and ASOVI patients, or between the three groups, except that the NIHSS score was significantly higher in the ASVI group than in the ASOVI and chronic CVT groups (medians 8, 0, and 0, respectively; P < 0.001). Of note, disorders of consciousness were only present in the ASVI patients (Table 1).

**Table 1:** Baseline characteristics of CVT patients in the different groups.

Characteristic	ASVI (n=30)	ASOVI (n=13)	Chronic CVT (n=9)	P value^†^	P value^*^
Age, year	33 (24-50.3)	32 (19.5-49.5)	38 (25-43)	0.552	0.807
Female	17 (56.7)	6 (46.2)	5 (55.6)	0.526	0.812
Genetic prothrombotic states	8 (26.7)	1 (7.7)	1 (11.1)	0.319	0.277
Acquired prothrombotic states	20 (66.7)	7 (53.8)	7 (77.8)	0.649	0.497
Hematologic diseases	4 (13.3)	2 (15.4)	2 (22.2)	1.000	0.811
Current craniocerebral infection	6 (20.0)	2 (15.4)	0 (0)	1.000	0.345
Non-infectious inflammatory disease	1 (3.3)	0 (0)	0 (0)	1.000	0.688
Interval from CVT onset to admission, days	9.5 (6.0-19.0)	15 (6.50-24.00)	40 (30-54)	0.361	<0.001
Disorders of consciousness	9 (30)	0	0	0.039	0.018
NIHSS	8 (0-16)	0 (0-0)	0 (0-0)	<0.001	<0.001

Values are expressed as medians [interquartile range, (IQR)] or n (%). CVT, cerebral venous thrombosis; ASVI, acute/subacute CVT with venous infarct; ASOVI, acute/subacute CVT without venous infarct; NIHSS, NIH Stroke Scale.

† P values for comparisons between ASVI and ASOVI groups.

* P values for comparisons between the three groups

### Increased serum hs-CRP and claudin-5 levels were associated with venous infarction in acute/subacute CVT patients

When the three groups were compared, serum hs-CRP was found to be significantly higher in the acute/subacute CVT groups than in the chronic CVT group (Table 2). Additionally, serum hs-CRP was significantly higher in the ASVI group than in the ASOVI group (medians 17.25 vs. 2.27 mg/l; respectively, P = 0.003) as well as claudin-5 (medians 2.80 mg/L vs. 2.50 mg/l, respectively; P = 0.039) (Table 2). Although there was no significant difference in the median levels of CSF occludin between the ASVI and ASOVI patients, it was detectable in 16 of the 30 (53.3%) ASVI patients while only 5 of the 13 (38.5%) ASOVI patients (P = 0.370). Additionally, some biomarkers of interest are not presented in Table 2: MMP-9 was not detected in the CSF of any patient and GFAP and S100B were undetectable in the serum of most patients, except for two ASVI patients with large-volume venous infarctions and brain herniations, with mean concentrations of 3.55 µg/l and 3.29 µg/l, respectively.

**Table 2:** Levels of biomarkers (mg/l) in serum and cerebrospinal fluid in the different CVT patient groups.

Protein	ASVI (n=30)	ASOVI (n=13)	Chronic CVT (n=9)	P value^†^	P value^*^
Serum hs-CRP	17.25 (5.02-32.07)	2.27 (0.60-10.74)	0.54 (0.15-2.87)	0.003	<0.001
Serum claudin-5	2.80 (2.37-3.70)	2.50 (1.94-2.70)	2.52 (1.80-3.01)	0.039	0.069
CSF claudin-5	0.49 (0.35-0.67)	0.4 (0.29-0.75)	0.387 (0.273-0.45)	0.492	0.197
Serum occludin	15.07 (12.23-17.71)	16.01 (14.34-17.38)	14.92 (12.22-16.84)	0.483	0.484
CSF occludin	0.71 (0.36-1.29)	0.19 (0.15-0.90)	-----	0.137	-----
Serum MMP-9	407.19 (103.72-775.42)	437.39 (273.69-902.59)	337.44 (105.57-746.16)	0.405	0.612

Values are expressed as the median (interquartile range) CVT, cerebral venous thrombosis; ASVI, acute/subacute CVT with venous infarct; ASOVI, acute/subacute CVT without venous infarct; CSF, cerebrospinal fluid; hs-CRP, high-sensitivity C-reactive protein; MMP-9, matrix metalloproteinase-9

† P values for comparison between the ASVI and ASOVI groups

* P values for comparisons between all three groups

**Figure 1.**
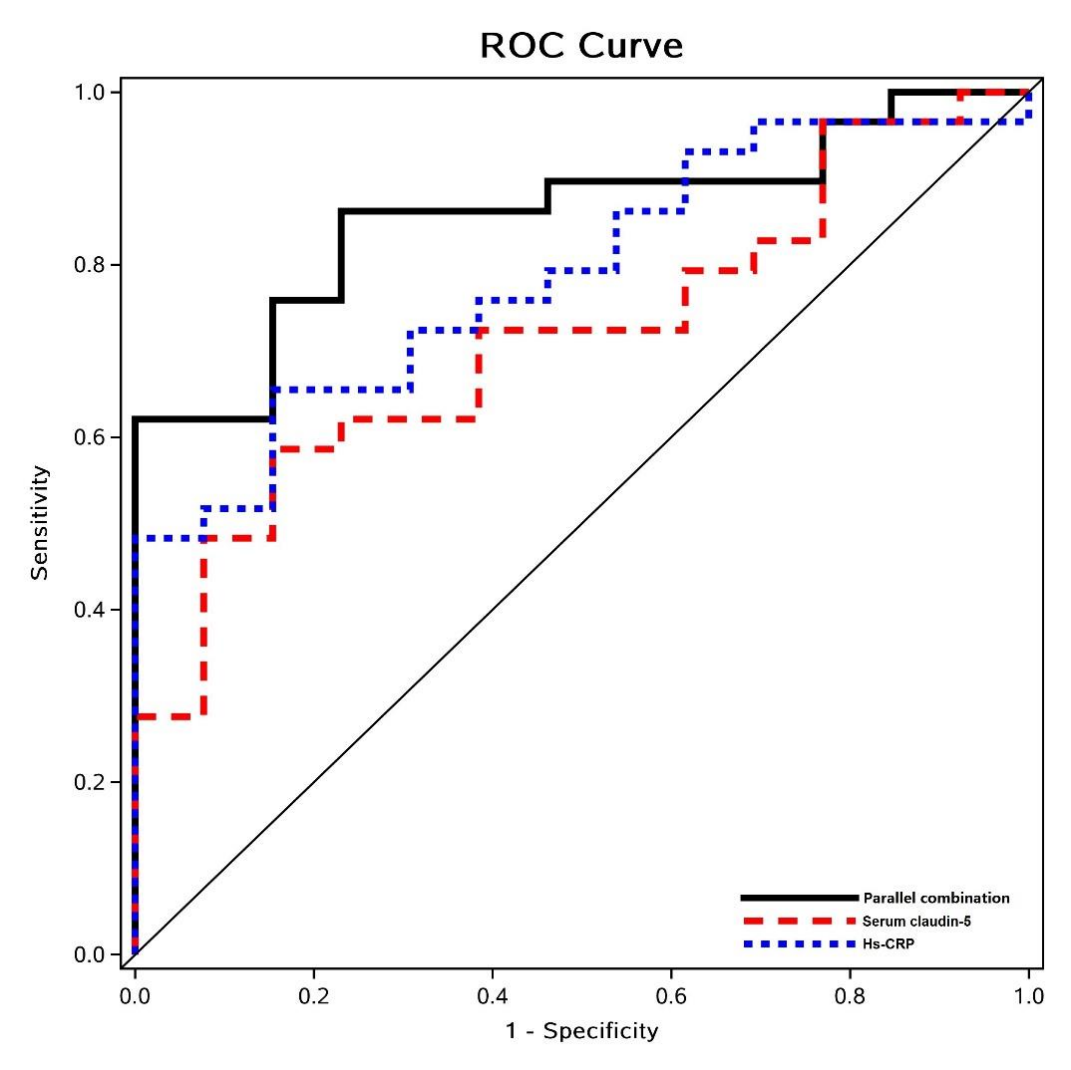
Assessing the capability of serum high-sensitivity C-reactive protein (hs-CRP) and serum claudin-5 individually and in combination to differentiate ASVI from ASOVI using receiver operating characteristic (ROC) analyses. Areas under the ROC curve (AUCs) were similar (P = 0.409) for hs-CRP [blue dashed line: 0.700, 95%CI (0.54-0.86), P = 0.027] and claudin-5 [red dashed line: 0.795, 95%CI (0.66-0.93), P = 0.003] in differentiating ASVI from ASOVI. The AUC was significantly larger for the combination of the two [black solid line: 0.862, 95%CI (0.75-0.97)] than for claudin-5 alone (P = 0.035).

The AUCs of ROC used to differentiate ASVI from ASOVI were: 0.700 [95%CI (0.54-0.86), P = 0.027] for serum claudin-5 alone, 0.795 [95%CI (0.66-0.93), P = 0.003] for hs-CRP alone, and 0.862 (95%CI 0.75-0.97, P < 0.001) for the combination of both. The AUC for the combination was significantly larger than that for claudin-5 alone (P = 0.035; Fig 1). To best differentiate ASVI from ASOVI, ROC analyses indicated a cut-off value of 2.74 mg/l for serum claudin-5 and 10.97 mg/l for serum hs-CRP. Based on the cut-off values noted above, we dichotomized hs-CRP and claudin-5 as binary variables.

Using the cut-off values above, the univariate logistic regression analyses of the 43 acute/subacute CVT patients showed that dichotomized hs-CRP [OR 10.45, 95%CI (1.92-56.64), P = 0.007] and claudin-5 [OR 7.19, 95%CI (1.35-38.24), P = 0.021] were significantly associated with the presence of venous infarction. When analyzed as continuous variables, hs-CRP and claudin-5 were also significantly associated with venous infarction in acute/subacute CVT patients (Table 3).

### Serum hs-CRP, but not claudin-5, was significantly associated with NIHSS on admission

In acute/subacute CVT patients, serum hs-CRP was positively and linearly associated with the NIHSS [*r* = 0.710, 95%CI (0.533-0.824), P < 0.001] on admission. In contrast, no significant linear association was found between serum claudin-5 and the NIHSS [*r* = 0.133, 95%CI (-0.223-0.401), P = 0.469].

## DISCUSSION

To date, little is known about reliable biomarkers associated with venous infarction in the setting of CVT. In this study, we assessed the levels of biomarkers related to BBB disruption and inflammation (Hs-CRP) in serum and CSF and their associations with venous infarction in a cross-sectional study of 52 patients with CVT. We found that serum hs-CRP was significantly higher in acute/subacute than in chronic CVT patients. Additionally, higher levels of serum claudin-5and hs-CRP were associated with venous infarction in acute/subacute CVT patients, with moderate-to-good predictive values of venous infarction by ROC analyses (AUCs of 0.700 and 0.795, respectively). Regarding the severity of clinical symptoms in acute/subacute CVT patients, only serum hs-CRP was positively and linearly associated with the NIHSS on admission.

**Table 3:** Univariate regression analyses for association between patient characteristics and biomarkers with venous infarction in acute/subacute CVT patients.

Variables	OR (95%CI)	P value
Age	1.02 (0.97-1.07)	0.491
Sex	0.66 (0.18-2.42)	0.527
Infection	1.38 (0.24-7.93)	0.722
Acquired prothrombotic states	1.71 (0.45-6.47)	0.427
Genetic thrombotic states	4.36 (0.49-39.17)	0.188
Serum hs-CRP		
Dichotomized by 2.74 mg/l	10.45 (1.92-56.64)	0.007
Continuous variable	1.12 (1.02-1.22)	0.016
Serum claudin-5		
Dichotomized by 10.97 mg/l	7.19 (1.35-38.24)	0.021
Continuous variable	1.001 (1-1.002)	0.050

CI, confidence interval; CVT, cerebral venous thrombosis; hs-CRP, high-sensitivity C-reactive protein; OR, odds ratio

A previous study has indicated that increased serum occludin levels are a potential marker of BBB damage during ischemia and reperfusion following an arterial ischemic stroke [26]. However, in this study, we found no significant association between serum/CSF occludin and venous infarction. Instead, we did identify a significant association between serum claudin-5 levels and venous infarction. This difference in findings may be partly due to differences in BBB damage between arterial ischemic stroke and venous infarction. Namely, in arterial ischemic stroke, BBB damage is mainly induced via cellular ischemic necrosis while venous infarction is associated with increased BBB permeability due to potentially increased venular and capillary pressure [6]. Claudin-5 has been indicated as a key factor involved in the endothelial permeability of the BBB [10,27] and a structural component of TJs [28]. Moreover, occludin (65 kDa) is three times larger than claudin-5 (20-24 kDa) [28]; therefore, BBB dysfunction related to venous infarction may more easily lead to the leakage of smaller proteins than that of larger proteins. No significant association was identified between the CSF claudin-5 level and the presence of venous infarction in this study, which suggests that claudin-5 might be released primarily into the bloodstream rather than the CSF after BBB disruption caused by venous infarction. In summary, the increase in serum claudin-5 may reflect the extent of BBB damage during venous infarction in acute/subacute CVT, based on current findings.

A recent meta-analysis demonstrated that MMP-9 had a high predictive value for hemorrhagic transformation after acute arterial ischemic stroke [29]. Although venous parenchymal hemorrhage is commonly seen in patients with venous infarction, serum MMP-9 levels were not significantly different between ASVI and ASOVI patients in the current cohort. Arterial infarction can lead to significant hypoxia in ischemic regions, and hypoxia increases the expression of MMP-9 [30]; however, a hypoxic state in venous infarction might not be as significant as that in arterial infarction, which may explain the current finding that MMP-9 was not significantly associated with venous infarction.

The levels of serum GFAP and S100B were also tested in each CVT group. Both were detected in only two ASVI patients with large-area venous infarction and brain herniation. Thus, the two proteins might only be released after significantly injured brain tissue due to severe venous infarction. However, we did not investigate the levels of GFAP and S100B in the CSF, which could be explored in further studies.

Previous studies have suggested significant inflammation in arterial atherothrombosis [31] and venous thrombus formation [18,32,33]. Hs-CRP, as a reliable biomarker for systemic inflammation, has been associated with cardiovascular diseases [34]. Ischemic stroke and myocardial infarction are well known to show inflammatory pathways in the acute and subacute stages, with elevated CRP levels. CRP has also been reported to be correlated with stroke severity as measured by the NIHSS [35,36]. A recent experimental study has revealed that CVT-induced BBB disruption and venous infarction may rely on the infiltration of inflammatory cells, which contribute to the edemagenic responses to CVT [20]. In this study, we found that serum hs-CRP was significantly associated with venous infarction in acute/subacute CVT patients. Moreover, serum hs-CRP levels were positively correlated with the NIHSS on admission, which corroborates previous findings and further emphasizes the significant role of inflammation in BBB disruption related to venous infarction. However, CVT shares risk factors with venous thromboembolism (e.g., deep vein thrombosis or pulmonary embolism), and inflammation is a common feature of several risk factors (e.g., cancer, infection, autoimmune conditions, trauma, surgery, pregnancy) for venous thromboembolism [37]. Inflammation can disrupt the hemostatic system, promoting a pro-thrombotic state [38]. However, due to the limitations of the study design, it is uncertain whether inflammation, represented by hs-CRP, is the causative agent of venous infarction or a response to venous infarction. Since inflammation and hemostasis are interrelated pathophysiologic processes that considerably influence each other [39], the relationship may be bidirectional. Further longitudinal studies with serial assessments of hs-CRP levels in CVT patients may provide valuable information regarding the causative, reactive, or bidirectional relationship between inflammation and venous infarction in such patients.

Our study had some significant strengths. First, both CSF and blood samples were collected on admission from all patients, which was less common in previous studies because of the low incidence of CVT. Additionally, despite the small sample size, the study was larger than most previous clinical studies on CVT. This study also had limitations. This was a cross-sectional study where we investigated the associations between the biomarkers and venous infarction based only on one test, and we did not follow up on the patients to determine long-term outcomes. Moreover, we did not differentiate between acute and subacute CVT patients in the analyses. This all limits the identification of time-specific biomarkers, their evolution over time, and their impact on long-term clinical outcomes. Future longitudinal studies with more accurate CVT staging and serial assessments of these biomarkers are recommended to further illustrate these relationships.

### Conclusion

In summary, the current study suggests that serum hs-CRP and claudin-5 might be significantly associated with venous infarction in acute/subacute CVT patients. Hence, venous infarction is not only associated with BBB disruption but also possibly with inflammation. Moreover, serum hs-CRP levels might reflect the severity of clinical symptoms in patients with acute/subacute CVT. These findings suggest that the inflammatory response might be involved in the pathogenesis of venous infarction in CVT patients. Therefore, anti-inflammatory treatments may be a promising therapeutic strategy for acute/subacute CVT with venous infarction. Future longitudinal studies with a larger sample size and serial assessments of these biomarkers are needed to corroborate these findings.
